# Inductive crystallization effect of atomic-layer-deposited Hf_0.5_Zr_0.5_O_2_ films for ferroelectric application

**DOI:** 10.1186/s11671-014-0711-4

**Published:** 2015-01-31

**Authors:** Xun Zhang, Lin Chen, Qing-Qing Sun, Lu-Hao Wang, Peng Zhou, Hong-Liang Lu, Peng-Fei Wang, Shi-Jin Ding, David Wei Zhang

**Affiliations:** State Key Laboratory of ASIC and System, School of Microelectronics, Fudan University, Shanghai, 200433 China

**Keywords:** Hf_0.5_Zr_0.5_O_2_, Ru, Ferroelectricity, Phase transition

## Abstract

Ferroelectric Hf_*x*_Zr_1-*x*_O_2_ thin films are considered promising candidates for future lead-free CMOS-compatible ferroelectric memory application. The inductive crystallization behaviors and the ferroelectric performance of Hf_0.5_Zr_0.5_O_2_ thin films prepared by atomic layer deposition were investigated. Inductive crystallization can be induced by the film growth condition and appropriate top electrode selection. In this work, a Ni/Hf_0.5_Zr_0.5_O_2_/Ru/Si stack annealed at 550°C for 30 s in N_2_ ambient after the Ni top electrode has been deposited was manufactured, and it shows the best ferroelectric hysteresis loop in the dielectric thickness of 25 nm, with a remanent polarization value of 6 μC/cm^2^ and a coercive field strength of 2.4 MV/cm measured at 10 kHz. Endurance, retention, and domain switching current characteristics were evaluated well for potential application in the field of ferroelectric field effect transistor (FeFET) and nonvolatile ferroelectric memories (FeRAM).

## Background

The well-known perovskite-based ferroelectric materials, such as lead zirconate titanate (PZT) and strontium bismuth tantalate (SBT), have been widely applied to solid-state devices. As a promising candidate for lower power, higher density nonvolatile memories, severe challenges should be solved before integrating such ferroelectric materials into conventional CMOS technology [1,2].

Currently, high-*k* materials, such as HfO_2_, ZrO_2_, Al_2_O_3_, Ta_2_O_5_, and ZnO, have received much interest as dielectrics in MOSFET structures, flash memory, RF, mixed signal ICs, and so on. Among the oxides mentioned, HfO_2_ and ZrO_2_ have been profoundly studied and have great potential to be put into applications [3]. Ferroelectric performance in SiO_2_-doped HfO_2_ with a remanent polarization (*P*_r_) above 10 μC/cm^2^ and a coercive field strength (*E*_c_) of 1 MV/cm was reported [1]. Ferroelectric Hf_0.5_Zr_0.5_O_2_ thin films for nonvolatile memory applications were recommended [4], which showed a *P*_r_ value of 16 μC/cm^2^ and *E*_c_ of 1 MV/cm. In addition, polarization measurements on Al:HfO_2_ based metal-insulator-metal capacitors also showed an antiferroelectric-to-ferroelectric transition depending on annealing conditions and aluminum content [5]. From the point of crystallography, previous experimental phenomena resulted from the special phase transformation that happened in the crystal structure of the annealed thin film, which caused a rare orthorhombic phase with the non-centrosymmetric space group P*bc*2_1_.

In this work, a ferroelectric metal-insulator-metal (MIM) capacitor utilizing the Hf_0.5_Zr_0.5_O_2_ layer was investigated. Ru was defined as the bottom electrode and the source of external stress to induce crystallization with the assistant effect of the Ni top electrode. In consideration of the precisely controlled factors of high-quality nanolaminate films, including inter-facial roughness, inter-diffusion between layers, layer-to-layer consistency, and conformality, the Hf_0.5_Zr_0.5_O_2_ layer was prepared by atomic layer deposition (ALD) because it is more powerful in preparing such multilayers than other techniques, which keeps the precursors separated during the reaction [6,7]. Physical and electrical characterization of the device performance was carried out including the analysis of crystallinity, polarization-voltage (*P*-*V*) hysteresis loops, endurance, retention, and domain switching current. Several material aspects of Hf_0.5_Zr_0.5_O_2_ thin films were discussed in order to give better insight into their ferroelectric properties and guidelines for transistor fabrication.

## Methods

Planar MIM capacitors were manufactured on Si substrates. The bottom electrode structure consisted of a 300-nm-thick SiO_2_ layer, 20-nm TiN, and 100-nm Ru at least for the purpose of induced crystallization. Hf_0.5_Zr_0.5_O_2_ thin films (10 to 25 nm) were grown in a single-wafer ALD reactor using tetrakis-(dimethylamino)-zirconium (TDMA-Zr) and tetrakis-(ethylmethylamino)-hafnium (TEMA-Hf) as precursors at 200°C. H_2_O was used as oxidant and nitrogen (N_2_) as purge and carrier gas. The 50-nm-thick Ni top electrode was deposited by sputtering technology under a power of 50 W in the cluster system. A subsequent rapid thermal processing (RTP) step was set at 823 K for 30 s in N_2_ ambient, resulting in crystallization.

Dielectric layer thickness was controlled accurately by adjusting the total cycle number and evaluated by spectroscopic ellipsometry. The stoichiometry was defined by varying the Zr/Hf + Zr ratio of the precursors and monitored by X-ray photoelectron spectroscopy (XPS) on samples without thermal treatment. Accordingly, X-ray diffraction (XRD) and transmission electron microscopy (TEM) were utilized to confirm the crystallinity of thin films. On the part of electrical characteristics, *P*-*V* measurements were performed to present MIM capacitors exhibiting ferroelectric polarization hysteresis at different test signals with the additional tests of endurance, retention, and domain switching current.

## Results and discussion

There are three kinds of crystallization phases of pure HfO_2_ under atmospheric pressure. Monoclinic phase (m-phase), which is stable at ordinary temperature, will turn to tetragonal phase (t-phase) at 2,000 K approximately, and cubic (c-phase) at 2,900 K. Besides, the orthorhombic phase (o-phase) may exist under high pressure. Ferroelectricity could not be found in HfO_2_-based materials prepared with normal methods, usually aimed at late-model high-*k* dielectric in research and application. So the experiment phenomena suggested that the t-phase possibly appears in high temperature and leads to a special transformation to the needed o-phase under stress during cooling in the HfO_2_-based thin film, leading to ferroelectricity.

There are two potential reasons for ferroelectric performance appearing in the thin film. One is doping in HfO_2_ thin films, which stabilizes the t-phase possibly during annealing and enhances the objective transformation during cooling. The other is external stress from electrodes, inhibiting the transformation from the t-phase to m-phase and resulting in a shearless transformation into the o-phase instead. In this work, Zr was chosen as the doping material and Ru and Ni as the bottom and top electrodes, respectively.

**Figure 1:**
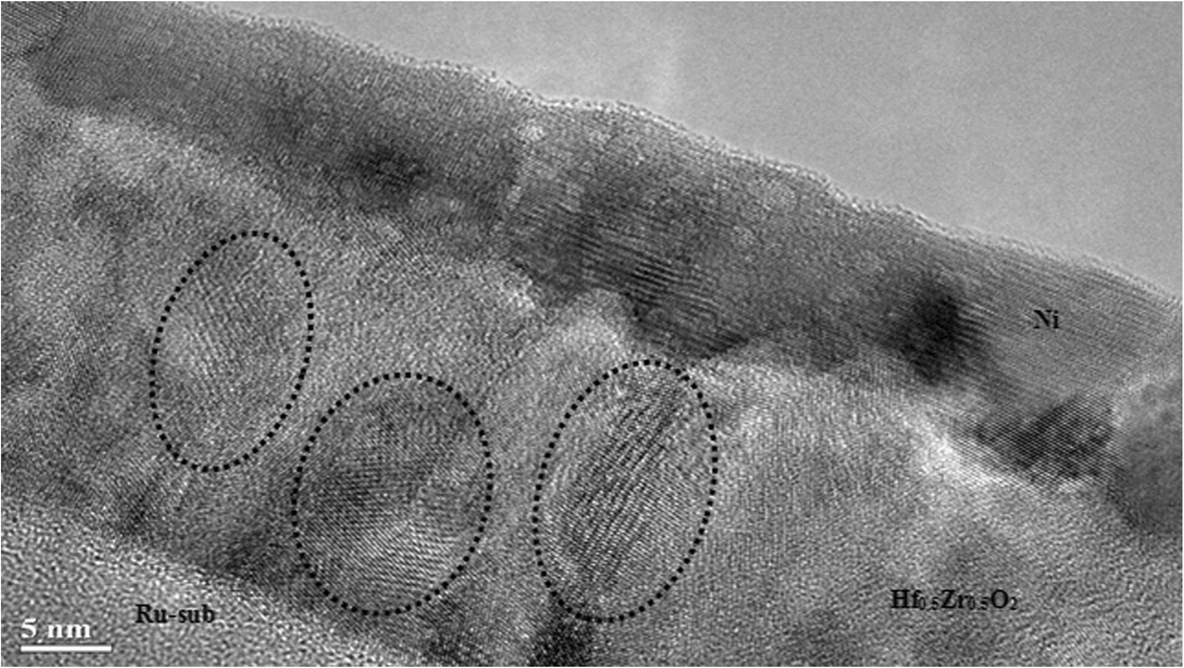
**A TEM cross section of the MIM capacitor with a 25**-**nm-thick Hf**_**0.5**_ **Zr**_**0.5**_ **O**_**2**_ **thin film.**

A TEM cross section of the MIM capacitor with a 25-nm-thick Hf_0.5_Zr_0.5_O_2_ thin film is shown in Figure 1. The Ni top electrode is formed at room temperature, followed by RTP at 823 K for 30 s in N_2_ ambient to crystallize the as-deposited amorphous Hf_0.5_Zr_0.5_O_2_ thin films, which enables CMOS-compatible device processing and also offers BEOL compatibility, compared with high heat budget of the SiO_2_-doped HfO_2_ system [1].

**Figure 2:**
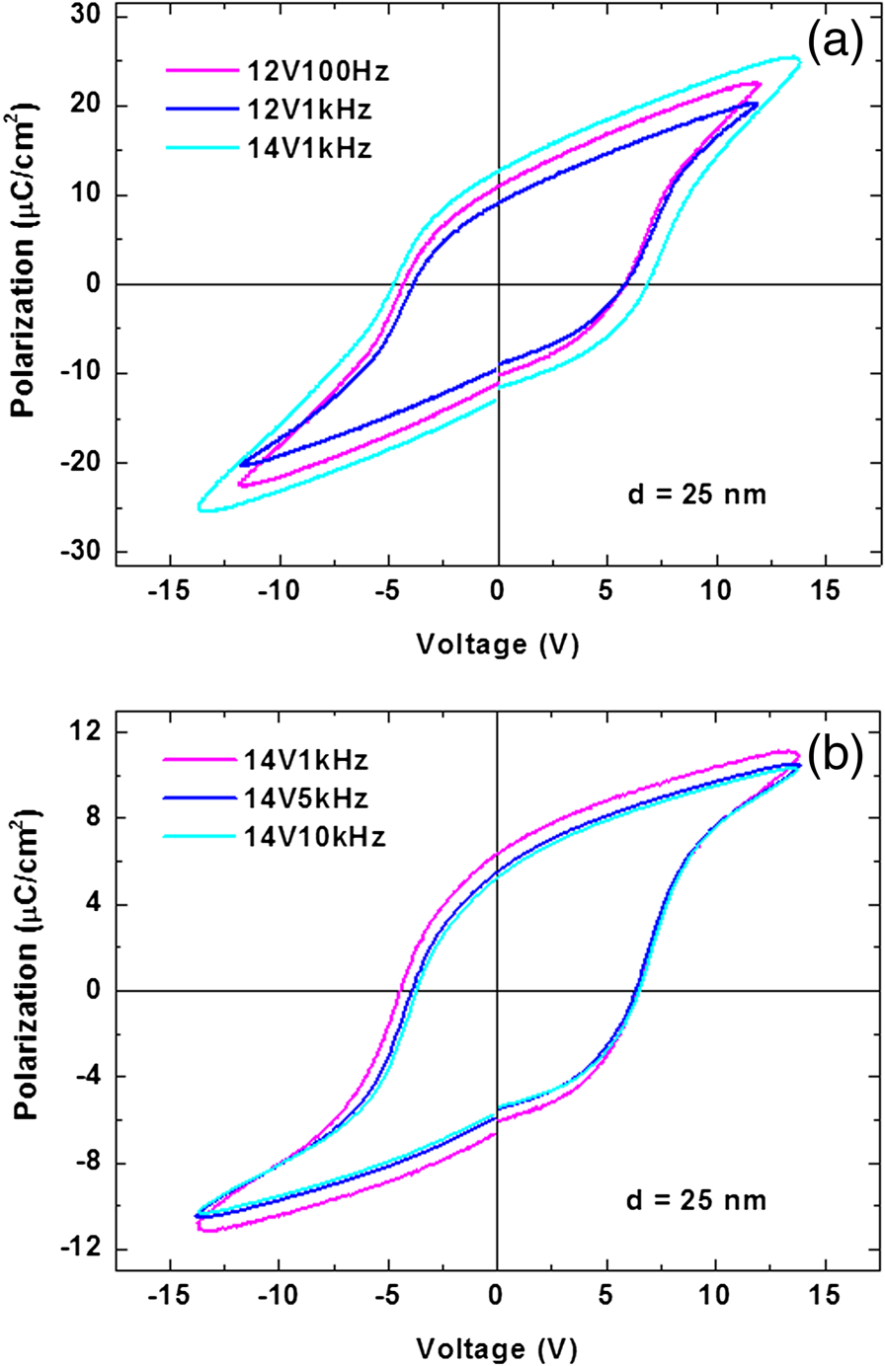
***P*** **-** ***V*** **hystereses of MIM capacitors with (a) uncapped and (b) capped structures at different signals.**

Evidence for the ferroelectric performance of the Ru/Hf_0.5_Zr_0.5_O_2_/Ni MIM capacitor is given by the *P*-*V* hysteresis measurement shown in Figure 2. In order to investigate the influence of mechanical encapsulation on HfZrO thin films, two samples were prepared with the only difference in the sequence of the deposition of the top electrode and the annealing of the thin film. If RTP is carried out before the deposition of the top electrode, the structure is known as uncapped (Figure 2a) and the opposite as capped (Figure 2b). Besides, from the results of our research, it is obvious that with the increase of thin film thickness, *P*_r_ raises while *E*_c_ is reduced. This is because of the larger quantity of domains [8]. So, 25 nm has been chosen as the thickness of the sample thin film.

It can be observed that the capped structure with a *P*_r_ value of about 6 μC/cm^2^ and *E*_c_ of 2.4 MV/cm at the peak voltage of 14 V expresses more stable and typical loops at variational frequencies compared with uncapping, especially in the range of high frequency, which means less leakage during the test of electrical characteristics and better ferroelectricity. Meanwhile, these loops are not closed because of the defects existing in the thin films, which makes enrichment of charges due to the positive electric field and forms the pinning effect decreasing the quantity of reversible domain when the extra electric field reverses.

**Figure 3:**
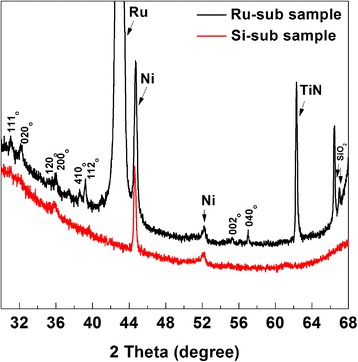
**The XRD spectra with Ru or Si as bottom electrode in** ***θ*** **-2** ***θ*** **mode.** Both of the samples have capped structure and Ni as the top electrode.

The choice of bottom electrode can affect the structure of the deposited ferroelectric thin film and its performance [9]. Figure 3 shows the contribution of the inductive crystallization effect of the Si/SiO_2_/TiN/Ru bottom electrode by XRD spectra, compared with a sample on Si-sub. Both of them have a capped structure. The reference powder pattern of the Hf_*x*_Zr_1-*x*_O_2_ system for the orthorhombic phase with space group P*bc*2_1_ [1,10] is calculated from the literature, whose estimated lattice parameters are 5.26 Å (a), 5.07 Å (b), and 5.08 Å (c).

In consideration of the effects of thermal stress and inevitable mechanical error during measurement, in spite of the typical phase mixture, the XRD results of the two samples has shown a diffraction pattern indicating a significant fraction of o-phase HfO_2_ which is similar for a rare orthorhombic phase with space group P*bc*2_1_, not centrosymmetric and therefore does not exclude ferroelectricity. It is obvious that the sample with the Ru bottom electrode shows more and stronger intensity of the o-phase peaks, which could be concluded that the grain size gets larger and the crystalline quality is improved, resulting in the increase of *P*_r_.

**Figure 4:**
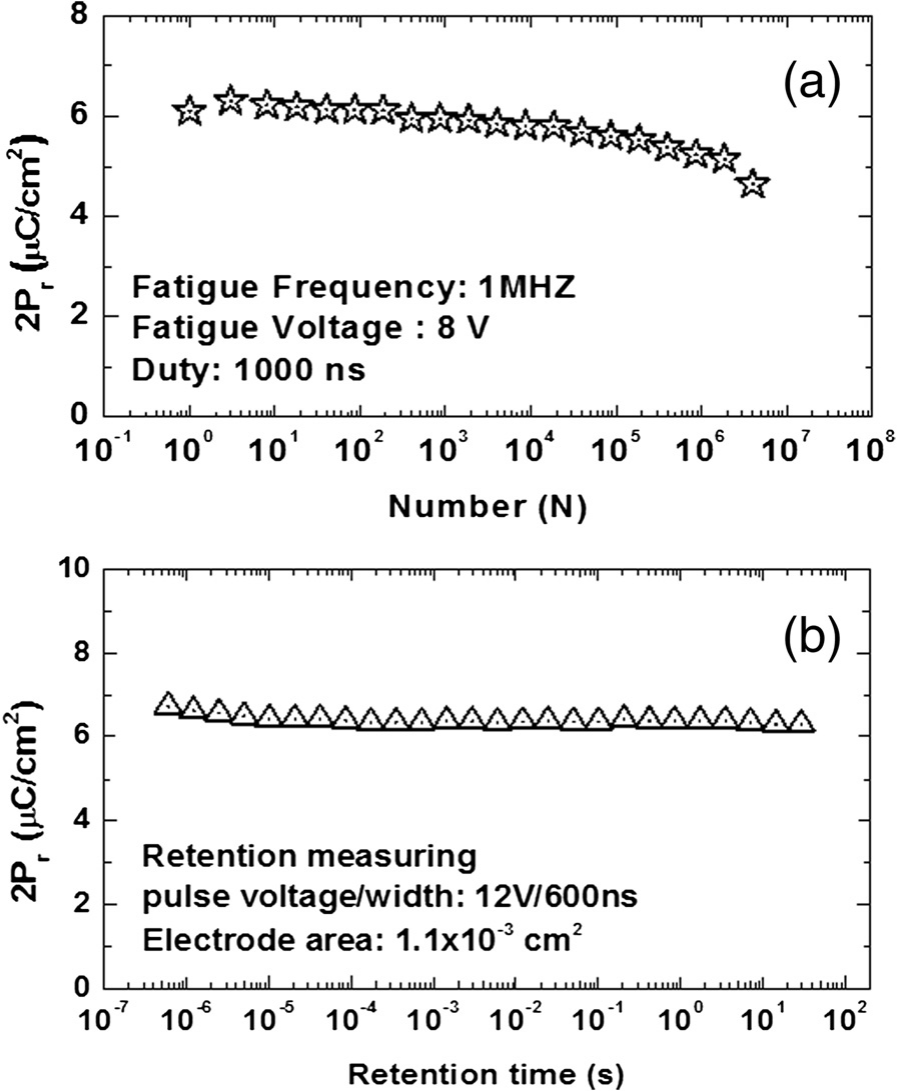
**Variations in the** ***P***_**r**_ **with (a) the number of work cycles, (b) retention time.**

For practical application in the field of ferroelectric field effect transistor (FeFET) or nonvolatile ferroelectric memories (FeRAM), HfO_2_-based ferroelectric thin films should achieve superior performance on endurance cycling and data retention. Figure 4 shows the change of *P*_r_ of the MIM capacitor with a 25-nm-thick thin film and Ni top electrode in capped crystallization as a function of the number of work cycles and retention time, respectively.

There is an evident effect of ferroelectric polarization fatigue during the endurance cycling test when *P*_r_ has reduced to about 5 μC/cm^2^ after 10^6^ work cycles, being inferior to traditional ferroelectric materials such as SBT, which will not give a clear indication of fatigue after 10^9^ times of switching. The phenomenon of fatigue in ferroelectric materials is caused by the pinning effect in the electric domain structure when the charged defects or oxygen vacancies are removed under the drive of external electric field and arrive at the surface of electrodes finally, which is called interface scenario admittedly [11].

On the other hand, the test performance of retention time is better. Because of a high Schottky barrier formed in the contact between the HfO_2_-based thin film and the Ni electrode, lesser intrinsic leakage current has been secured and enforced the optimization of retention of the MIM capacitor. In addition to the contact characteristic, appropriate bias voltage and residual injected interface charge can also improve the data retention significantly [12].

**Figure 5:**
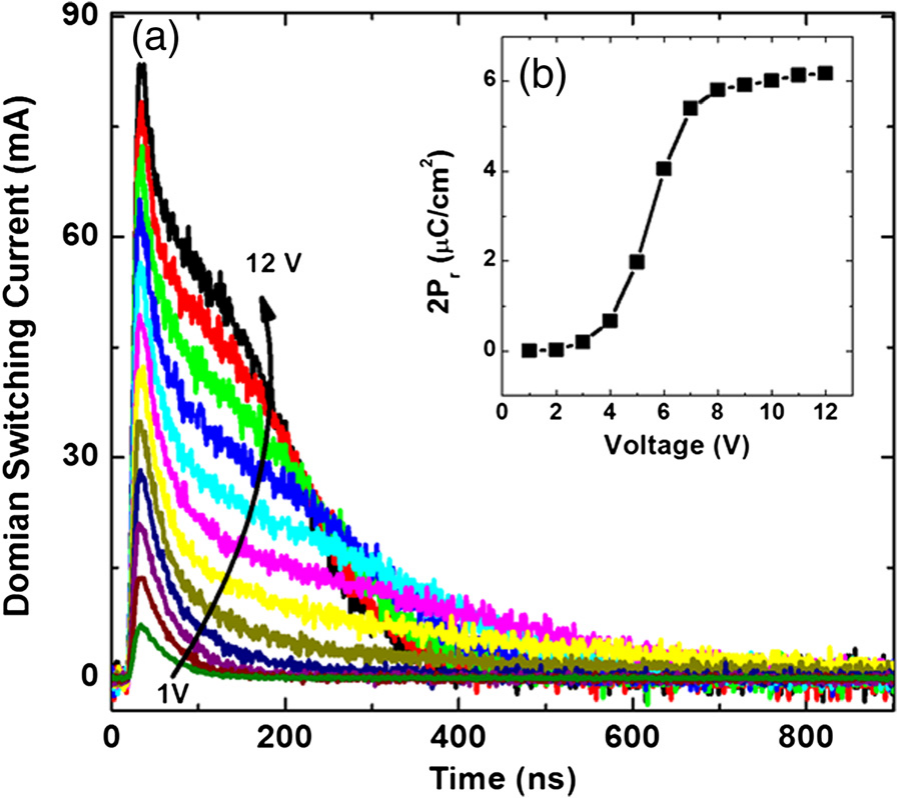
**Variations in the domain switching current and** ***P***_**r**_ **. (a)** Variations in the domain switching current as a function of the peak voltage and test time. **(b)** Variations in the *P*_r_ as a function of the peak voltage.

With the influence of external electric field, spontaneous polarization will alter its orientation, inducing the domain switching current (Figure 5). The peaks of current slightly increase, and the values of time to reach the peak gradually decrease with the increasing of electric field strength during the test, suggesting the higher speed of domain wall inversion. Combining with Figure 5b, it is clear that a high *P*_r_ gives rise to a high polarization reversal current. Through the test results, although the one structure presents the best ferroelectric hysteresis loop, its switching current has risen up to about 80 mA in the 12-V test voltage. Considering the electrode area of only 1.1 × 10^−3^ cm^2^, the severe phenomenon of leakage is clear, which needs improvements.

## Conclusions

Ferroelectric cells with the Ru bottom electrode and the o-phase 25-nm-thick Hf_0.5_Zr_0.5_O_2_ thin film provided the inductive crystallization of Hf_0.5_Zr_0.5_O_2_ conventional ferroelectric performance. Such devices yielded a *P*_r_ value of 6 μC/cm^2^ and *E*_c_ strength of 2.4 MV/cm measured at 10 kHz. Accordingly, electrical tests were utilized to obtain the characteristic features, such as low retention loss expected for FeFET and FeRAM. Further systematic research is necessary for promoting ferroelectric Hf_*x*_Zr_1-*x*_O_2_ as promising materials for lead-free FeFET memory technologies.
